# Extracellular Calcium Influx Pathways in Astrocyte Calcium Microdomain Physiology

**DOI:** 10.3390/biom11101467

**Published:** 2021-10-06

**Authors:** Noushin Ahmadpour, Meher Kantroo, Jillian L. Stobart

**Affiliations:** College of Pharmacy, Rady Faculty of Health Sciences, University of Manitoba, 750 McDermot Avenue, Winnipeg, MG R3E 0T5, Canada; ahmadpon@myumanitoba.ca (N.A.); kantroom@myumanitoba.ca (M.K.)

**Keywords:** astrocytes, Ca^2+^ transients, ion influx, ionotropic receptors, Ca^2+^ channels, sodium-calcium exchanger, gliotransmission

## Abstract

Astrocytes are complex glial cells that play many essential roles in the brain, including the fine-tuning of synaptic activity and blood flow. These roles are linked to fluctuations in intracellular Ca^2+^ within astrocytes. Recent advances in imaging techniques have identified localized Ca^2+^ transients within the fine processes of the astrocytic structure, which we term microdomain Ca^2+^ events. These Ca^2+^ transients are very diverse and occur under different conditions, including in the presence or absence of surrounding circuit activity. This complexity suggests that different signalling mechanisms mediate microdomain events which may then encode specific astrocyte functions from the modulation of synapses up to brain circuits and behaviour. Several recent studies have shown that a subset of astrocyte microdomain Ca^2+^ events occur rapidly following local neuronal circuit activity. In this review, we consider the physiological relevance of microdomain astrocyte Ca^2+^ signalling within brain circuits and outline possible pathways of extracellular Ca^2+^ influx through ionotropic receptors and other Ca^2+^ ion channels, which may contribute to astrocyte microdomain events with potentially fast dynamics.

## 1. Introduction

Astrocytes are brain glial cells that contact nearby neurons and enwrap blood vessels with their highly branched processes. Physiologically, astrocytes are critical for brain homeostasis [1]. They buffer extracellular ions [2], they remove and recycle neurotransmitters [3,4,5], and they supply neurons with energy substrates [6,7,8,9]. However, astrocytes also express a plethora of neurotransmitter receptors, ion channels, and metabolite transporters that respond to nearby neuronal activity and integrate astrocytes into neural networks [1]. Many of these receptors and ion channels induce transient increases in intracellular Ca^2+^ [10] that are required for various astrocyte functions, as discussed below [10,11,12,13,14,15]. Recently, localized Ca^2+^ transients in fine astrocytic structures, such as processes and endfeet around blood vessels, have been identified using genetically encoded Ca^2+^ indicators (GECIs), such as GCaMP6f [16,17,18,19,20,21,22,23,24,25]. Here, we refer to these small, localized Ca^2+^ transients as astrocyte microdomain Ca^2+^ events (MCEs).

Astrocyte MCEs are heterogenous; they vary in amplitude and duration, and occur within astrocytes at rest (i.e., in the absence of nearby synaptic activity) [17,18]. The dynamics of astrocyte Ca^2+^ transients are dictated by the resting, basal intracellular Ca^2+^ concentration [26], which is higher in fine processes compared to the soma [27]. The number of astrocyte MCEs, their volume, and their amplitude increases [17,18,19,28,29] following nearby neuronal responses evoked by physiological stimuli, such as whisker stimulation-induced somatosensory activation [17,18,30,31], visual stimulation of the visual cortex [29], or odor presentation in the olfactory bulb [28]. The majority of astrocyte somatic Ca^2+^ events [32,33,34] and MCEs [17,18] activated during local circuit activity have a delayed signal onset latency (for example: MCEs arise 5 s after the start of whisker stimulation). Compared to neuronal Ca^2+^ signal onset timescales (a few milliseconds after the start of stimulation), this astrocytic Ca^2+^ signalling was deemed too slow to modulate rapid processes such as synaptic activity or blood flow [32,33,34]. However, fast onset Ca^2+^ dynamics have recently been described within fine astrocyte structures in response to physiological stimuli in vivo [17,28,30,31,35]. In particular, a subset of astrocyte MCEs near the plasma membrane of astrocyte processes, have a fast signal onset that closely follows neuronal activity (within 100 ms) and are reproducibly evoked within the same regions during repeated whisker stimulation [17]. This suggests that astrocytes have the necessary temporal and spatial Ca^2+^ signalling to play a rapid role in fine-tuning circuits as discussed below.

## 2. Functional Roles of Astrocyte Microdomain Ca^2+^ Events

Astrocytes are active contributors to brain processes through the release of gliotransmitters or vasoactive molecules that modulate the nearby neuronal activity or blood flow [10,11,12]. The gliotransmitters released by astrocytes include glutamate [36], GABA [37,38], ATP [39,40], and possibly D-serine [41,42] (though this remains controversial, as there is evidence of D-serine release from neurons [43,44]). These molecules act on neuronal receptors or nearby astrocyte receptors as a form of glial communication [11]. The release of these molecules is Ca^2+^ dependent, suggesting that astrocyte Ca^2+^ events are a key component of bidirectional astrocyte-neuron interactions [11,19]. Specifically, MCEs may play a critical role in confined, localized delivery of gliotransmitters that influence local synaptic activity [39,40,45,46,47,48,49,50], and the recruitment of larger Ca^2+^ domains or more global astrocyte Ca^2+^ signals may modulate neuronal networks and dictate animal behaviour [51,52,53,54,55] as outlined more specifically below.

**Figure 1:**
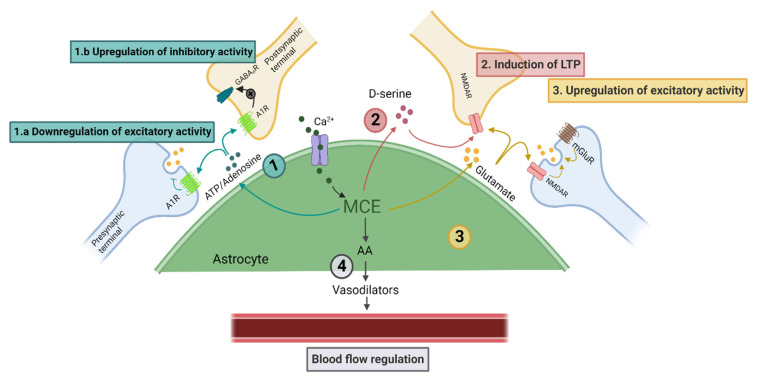
Examples of functional roles of astrocyte Ca^2+^ events. MCEs lead to gliotransmission: (1) ATP/adenosine a. downregulates the excitatory activity by activating presynaptic A1R [60] and b. upregulates inhibitory activity by activating postsynaptic A1R [40]. (2) D-serine enhances LTP via postsynaptic NMDARs [41]. (3) Glutamate released from astrocytes modulates pre- and post-synaptic neuronal glutamate receptors [36,50,56,57,59,61]. (4) In astrocyte endfeet, MCEs cause the production of arachidonic acid (AA) that is metabolized to vasodilative components, such as prostaglandins, and contribute to regulation of cerebral blood flow [12].

At the synaptic level, astrocyte Ca^2+^ signalling and gliotransmitter release influences basal synaptic activity, excitatory and inhibitory neurotransmission, and synaptic plasticity (Figure 1) [36,39,40,41,45,50,56,57,58,59]. Some specific examples include, first, astrocytes modulate basal synaptic transmission in the hippocampus [39,45,60] through adenosine that is likely produced from the metabolism of astrocyte ATP released during gliotransmission. Adenosine activates presynaptic A2A [39] or A1 receptors [60] to encourage or reduce neurotransmitter release, respectively. Second, hippocampal pyramidal neuron inhibition is enhanced by astrocyte ATP/adenosine gliotransmission at inhibitory interneuron synapses [40]. Third, glutamate released from astrocytes at excitatory synapses can increase synaptic release [59], boost synaptic strength [57], and elevate neuronal synchrony [36]. Finally, astrocyte glutamate [50,56,61] and D-serine [41,62] also contribute to long-term potentiation (LTP) and long-term depression (LTD) that are important for synaptic plasticity. This may include cholinergic-induced synaptic plasticity following activation of the nucleus basalis [50,63,64]. 

These examples highlight the diversity of astrocyte-neuron interactions at different synapses and via distinct gliotransmitters; however, a link between localized MCEs and gliotransmission has not been proven. The majority of these studies described above demonstrated a requirement of astrocyte Ca^2+^ signalling for the modulation of synaptic processes by utilizing Ca^2+^ chelator BAPTA [39,40,45,56,57] or clamping intracellular Ca^2+^ levels [41]. These approaches effectively silence all astrocytic intracellular Ca^2+^ events from microdomains to somatic transients to global Ca^2+^ waves, irrespective of their cellular location. Future studies that decode the effect of MCEs in astrocytic processes by targeting specific pathways will help to better disentangle the roles of astrocytes in gliotransmission and neuronal modulation.

In addition to gliotransmission, Ca^2+^ events can induce morphological remodeling of fine astrocytic processes at synapses [65,66,67]. This has the potential to change their synaptic coverage, affecting gliotransmission and synaptic function [68], and suggests that localized astrocytic MCEs within perisynaptic processes may regulate the stability of individual synapses.

During periods of neuronal activity, astrocyte Ca^2+^ signalling increases both in the number of MCEs within each cell as well as an enlargement of the MCE spatial area [17,18,19,28,29,30,31]. It has been suggested that a scaling of astrocyte Ca^2+^ signalling may induce heterosynaptic modulation where astrocytes integrate information from multiple synapses to influence additional neighbouring connections, or modulate an entire territory or neuronal network depending on the level of the evoked Ca^2+^ response [11]. For example, astrocytes play a regulatory role in neocortical slow oscillations that underlie resting brain waves [69,70], since Ca^2+^ signalling in astrocytes precedes a shift to slow-wave oscillations [70] and induces cortical UP states, where multiple neurons are synchronized [69]. Additionally, multiple studies have shown that robust, global Ca^2+^ events in astrocytes occur when norepinephrine is released from the locus coeruleus [15,24,33,71,72], suggesting that astrocytes have an important role in network modulation during arousal. Astrocytes have also been linked to animal behaviour, since increased Ca^2+^ in the hippocampus enhances memory formation [52], while mouse models with reduced astrocyte Ca^2+^ events (by targeting specific pathways in different brain regions) have repetitive [53], depressive [54], or autistic-like behaviours [55]. Thus, astrocytes may “sense” nearby neuronal activity through Ca^2+^ events that locally regulate circuit activity, modulate the processing of information in large networks and impact animal behaviour. Fast onset MCEs evoked by neuronal activity could be of critical importance for rapidly tuning changes at single synapses that amount to alterations in activity over larger circuits. Again, future studies specifically targeting pathways that contribute directly to astrocyte MCEs will help to link MCEs to the modulation of single synapses, but will also help determine how the scaling of astrocyte Ca^2+^ signalling and the recruitment of MCEs influence larger neuronal networks and behaviour.

Astrocytes may also regulate local blood flow through the Ca^2+^-dependent release of vasoactive molecules, such as arachidonic acid metabolites (Figure 1) [12]. This is important for tonic blood vessel tone [13], particularly during vasomotion [73]. However, a fast, dynamic role for astrocytes in regulating vasodilation during neurovascular coupling remains controversial. Early studies in brain slices ex vivo linked astrocyte Ca^2+^ to changes in vascular tone [12,74,75,76,77], but this has not translated to in vivo experiments where astrocyte Ca^2+^ events, particularly in endfeet microdomains, may [28,30,31] or may not [32,72,78] rapidly precede vasodilatory responses during neurovascular coupling. Several of these recent in vivo studies suggest astrocyte Ca^2+^ events are not essential for vasodilation [32,72,79]; however, when astrocyte endfoot Ca^2+^ signals are evoked by brief, local circuit activity, the magnitude of the hemodynamic response is enhanced [79]. During prolonged sensory stimulation [79] or the postictal epileptic period [80], slow, sustained astrocyte Ca^2+^ signals are induced, which correlate with vasoconstriction [81]. Therefore, while astrocytes and MCEs may not rapidly evoke blood flow changes during neurovascular coupling, they provide important, complex homeostatic and modulatory effects on blood flow that are relevant for both vasodilation and vasoconstriction at rest and during periods of brain activity [82].

## 3. Pathways Underlying Fast Astrocyte MCEs

A number of mechanisms are known to contribute to localized astrocyte MCEs [10,15,20,25]. Spontaneous astrocyte MCEs that occur in the absence of synaptic activity have been shown to be mediated by mitochondrial Ca^2+^ release [14] via the opening of mitochondrial permeability transition pore [15] and by extracellular Ca^2+^ influx through transient receptor potential cation channel A1 (TRPA1) [20,25]. It should be noted that other TRP channels such as TRPV1, TRPV4, TRPC1, TRPC3, TRPC4, and TRPC5 may also mediate Ca^2+^ influx in astrocytes [83,84,85,86,87,88], but there is limited evidence that these channels are directly activated during synaptic transmission.

**Figure 2:**
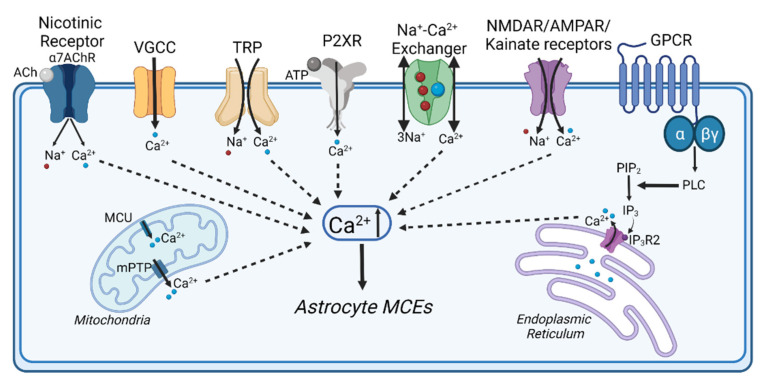
Astrocyte Ca^2+^ pathways activated during synaptic transmission. This diagram highlights the pathways that involve extracellular Ca^2+^ influx as discussed in this review.

The most extensively studied astrocyte pathway that contributes to Ca^2+^ events is the release of Ca^2+^ from the endoplasmic reticulum following inositol-1,4,5-trisphosphate receptor (IP_3_R) and upstream Gq-G-protein coupled receptor (GPCR) activation (Figure 2) [1]. This mechanism has been targeted in astrocytes using an IP_3_R2 knockout mouse [17,24,32,55,89,90], since IP_3_R2 is believed to be the principal isoform in astrocytes [91]. Knockout of endoplasmic reticulum IP_3_R2 reduces the number of astrocyte MCEs [17,18,24], but does not prevent increased astrocyte MCE responses in fine processes to arousal [24] or sensory stimulation [18], nor does it reduce the number of fast onset MCEs evoked by nearby synaptic activity [17]. Metabotropic glutamate receptors (mGluRs) were one of the first Gq-GPCR pathways found to elevate Ca^2+^ in astrocytes [77,92,93]. However, these receptors are potentially more important during development because mature, adult astrocytes have low mGluR mRNA expression [94] and reduced calcium responses to mGluR agonists [95], though this does not exclude mGluR expression and signalling in the fine processes of adult astrocytes [10,96]. Several other GPCR pathways that evoke IP_3_ signalling in astrocytes are activated by neuromodulators, such as norepinephrine and acetylcholine. These cause astrocyte Ca^2+^ transients during behavioural arousal states [17,24,71,72], but contribute more to large, delayed onset MCEs [17,24]. This suggests that fast onset MCEs are mediated by mechanisms other than GPCR activity, such as extracellular Ca^2+^ influx. Here, we discuss key pathways for rapid astrocyte Ca^2+^ influx through ionotropic receptors and ion channels that are activated during neurotransmission and may play important physiological roles in brain circuits (Figure 2).

### 3.1. Ionotropic Glutamate Receptors (NMDA, AMPA, and Kainate Receptors)

#### 3.1.1. Astrocyte iGluR Expression

Ionotropic glutamate receptors (iGluRs) are ligand-gated ion channels that conduct cations (Na^+^, Ca^2+^ and K^+^) when activated by synaptic glutamate (Figure 2), and this mediates fast excitatory synaptic transmission. Based on their selective agonists, iGluRs are categorized into three classes, including α-amino-3-hydroxy-5-methyl-4-isoxazolepropionic acid (AMPA) receptors, kainate receptors, and *N*-methyl-D-aspartate (NMDA) receptors [97]. AMPA receptors are tetramers formed from four possible subunits (GluA1-GluA4), which dictate the functional properties of the receptor, including their calcium permeability [98]. These receptors also generally have rapid deactivation kinetics [99]. Classical NMDA receptors are hetero-tetramers formed from two GluN1 subunits and two GluN2 subunits (of four possible types, A—D) [100]. There are also less-common GluN3A and 3B subunits, which can join GluN1 and GluN2 to form “non-conventional” receptors or possibly trimeric GluN1/GluN3 receptors [101,102]. The GluN2 subunit composition of conventional NMDA receptors confers functional properties such as a sensitivity to blockade by Mg^2+^, deactivation kinetics, and Ca^2+^ permeability [100]. Receptors containing GluN2C/2D are insensitive to blockade by Mg^2+^ and therefore do not require membrane depolarization to be activated. These subunits are also less permeable to Ca^2+^ than GluN2A/2B receptors, and they have slower deactivation kinetics [100].

Astrocytes express the genes of iGluRs, albeit at lower levels than neurons. All four AMPA receptor subunits (GluA1-GluA4) have been detected in astrocytes [1,103], although with some regional differences in expression [104,105]. For example, GluA1 and GluA4 are the most common subunits in cortical astrocytes and potentially localize to astrocyte processes [104]. Hippocampal astrocytes may also express GluA2 [106], which reduces calcium permeability through heteromeric receptors [105]. At early developmental stages (before postnatal day 5), astrocyte AMPA receptors deactivate slower than more mature stages (over postnatal day 10) [107]. This suggests that AMPA receptors on mature astrocytes may contribute to brief Ca^2+^ transients before deactivation. At the mRNA and protein level, NMDA receptor subunits GluN1 and GluN2A/B have been identified in astrocytes [1]. However, pharmacological studies suggest that functional NMDAR in astrocytes contain GluN2C/D and are most probably a heteromeric composition of GluN1, GluN2C/D, and GluN3 [108,109,110]. This explains the low sensitivity of astrocyte NMDA receptors to blockage by Mg^2+^ within the channel pore, and suggests that these receptors are resistant to deactivation, but have reduced Ca^2+^ permeability (compared to GluN2A/B receptors). There is evidence of astrocyte kainate receptor subunit expression at the mRNA and protein levels [111,112]; however, the functionality of these receptors remains controversial [1,113,114,115,116,117].

While astrocytes express iGluRs, the functionality of these receptors, particularly regarding Ca^2+^ permeability and their contribution to Ca^2+^ signalling, has been controversial. Early Ca^2+^ imaging studies were conducted in primary astrocyte cultures (Table 1), with several possible issues that could influence the interpretation of the results. First, some of these studies failed to detect NMDA-induced Ca^2+^ transients in astrocytes [113,114,115,118], but they used 100 μM NMDA, which is over the toxicity concentration threshold (50 μM) [119,120]. When 20 μM NMDA was applied, astrocytic Ca^2+^ responses were evoked [121]. Second, quisqualate (QA) was used as an agonist in some studies to identify functional AMPA and kainate- iGluRs [113,114,115,122]. However, quisqualate is not an iGluR-specific agonist and can activate metabotropic glutamate receptor I (mGluR I), which may have contributed to the mixed findings that QA-evoked Ca^2+^ responses have an internal Ca^2+^ store component [114,115,122]. Application of more specific agonists, such as AMPA, confirmed the presence of functional AMPARs on cultured hippocampal, cortical, and cerebellar astrocytes [122,123] as well as astrocytes in isolated optic nerve [124]. Third, astrocytes were cultured from different brain regions including the cortex, cerebellum, and hippocampus in these studies. Recent evidence suggests that there are regional iGluR expression differences in astrocytes [104,105,108,109,110], which may alter the Ca^2+^ permeability of the receptor and make it harder to compare results between studies [105,125]. Finally, the main limitation of astrocyte culture studies is that cells are isolated from neonatal animals and maintained for weeks in culture before the experiment. Thus, cultured cells may not reflect the mechanisms and receptor-activated effects of in situ astrocytes [126].

More recent studies have examined iGluR-mediated Ca^2+^ dynamics in ex vivo brain slices or acutely isolated astrocytes (Table 2). These preparations used astrocytes that are similar to in situ cells and often combined Ca^2+^ imaging with electrophysiological recordings. The majority of these studies reported a contribution of AMPA [28,108,124,127] and/or NMDA receptor [93,108,109,110,117,124,128,129,130,131] signalling to astrocyte Ca^2+^ transients; however, there are a few points to note when considering this work. First, the use of iGluR pharmacology can make it difficult to disentangle the activity of astrocyte receptors from neuronal receptors, particularly when the drugs are bath applied to brain slices where both cell populations are present (Table 2). Studies that have patch-clamped astrocytes and applied iGluR agonists or antagonists directly through the patch pipette have provided more convincing evidence of functional astrocyte AMPA and NMDA receptors that induce cell depolarization and Ca^2+^ events [108,109,128,129,131,132]. Second, as mentioned above, astrocyte iGluR ion fluxes may vary between different brain regions. For example, astrocyte NMDAR in the hippocampus and cortex permits different levels of Na^+^ influx [125]. Also, Na^+^ influx through cortical astrocyte NMDAR, induces reversal of the Na^+^/Ca^2+^ exchanger that can further evoke Ca^2+^ events, as described below [125,133]. This raises the interesting possibility that astrocyte iGluR signalling is fine-tuned to modulate the needs of the local circuit, but it makes it challenging to compare and interpret results from studies of astrocytes in different brain regions (Table 2). Finally, both juvenile [28,92,93,124,127,131,134] and adult animals [108,109,110,117,129,130] have been used to study astrocyte iGluR receptors ex vivo and this may provide conflicting results regarding astrocyte NMDARs because the subunit composition and their characteristics change during development [107,132]. Specifically, AMPAR deactivates faster in more mature astrocytes [107], while adult mice over 3 months of age display larger NMDAR-mediated currents and calcium transients than younger animals [132].

Although iGluR agonists evoke Ca^2+^ transients in astrocytes in culture and brain slices, most studies have focussed on somatic Ca^2+^ events. It is still unclear if these receptors contribute to astrocyte MCEs within fine processes, particularly during local circuit activity. Several studies have distinguished between Ca^2+^ responses in different cellular compartments (processes versus soma) by combining Ca^2+^ imaging dyes with GFAP-eGFP transgenic mice to better label astrocytes [110,128,129]. However, GECIs are now the most reliable way to detect astrocyte Ca^2+^ events in fine structures. Using GCaMP3 and GCaMP6f, Haustein et al. [135] showed that NMDAR blocker, D-AP5, did not change spontaneous astrocyte MCEs in the hippocampus, which indicates that astrocyte NMDAR may only be activated during nearby synaptic activity. Topical superfusion of AMPA or NMDA receptor antagonists on the brain, significantly reduced slow-onset MCEs in astrocyte endfeet evoked by whisker-stimulation, suggesting that iGluR signalling contributes to these Ca^2+^ events [72]. In similar studies, fast onset MCEs in astrocyte fine processes and endfeet were identified in response to stimulation of the contralateral ramus infraorbitalis of the trigeminal nerve [30,31], which is physiologically similar to sensory stimulation. The fast astrocyte Ca^2+^ responses happened on the same time scale as neurons and preceded local vasodilation. Blockers for AMPA or NMDA receptors were applied directly to the brain and both drugs reduced fast Ca^2+^ events in astrocyte processes, but only CNQX reduced fast Ca^2+^ events in endfeet [30]. This suggests that iGluR signalling may mediate rapid astrocyte MCEs that have the capacity to contribute to blood flow. The main drawback of all these studies of iGluRs and MCEs is that the pharmacological approaches employed likely affected both neuron and astrocyte receptors [28,30], making it unclear whether the drugs have direct effects on astrocyte iGluRs or if the impact on MCE activity was merely caused by decreased neuronal activity. Future work specifically targeting astrocyte iGluRs by genetic approaches will help to tease apart a role for these receptors in astrocyte MCE signalling, including fast onset events.

#### 3.1.2. Functional Roles of Astrocyte iGluRs

While it is clear that AMPA receptor activation can cause an elevation in astrocyte Ca^2+^ in the soma, limited studies have found a functional role for astrocyte AMPAR. In the cerebellum, Bergmann glia astrocytes express the GluA1 and GluA4 subunits [136]. When Bergmann glial AMPAR activity is inhibited by (a) expression of the GluA2 subunit that renders AMPAR Ca^2+^ impermeable [137] or (b) the conditional knockout of GluA1 and GluA4 [136], structural changes occur within the molecular layer of the cerebellum. Glial fine processes retract from Purkinje cell dendritic spines, which leads to delayed glutamate uptake at synapses [137] and deficits in fine motor control [136]. Clearly, Bergmann glia AMPAR are essential components of cerebellar circuits. Further work is required to determine the functional relevance of astrocyte AMPA receptors in other circuits (cortex, hippocampus, etc.), but also to determine if the fast deactivation kinetics of AMPA receptors limit their contribution to astrocyte MCEs and other signalling.

**Figure 3:**
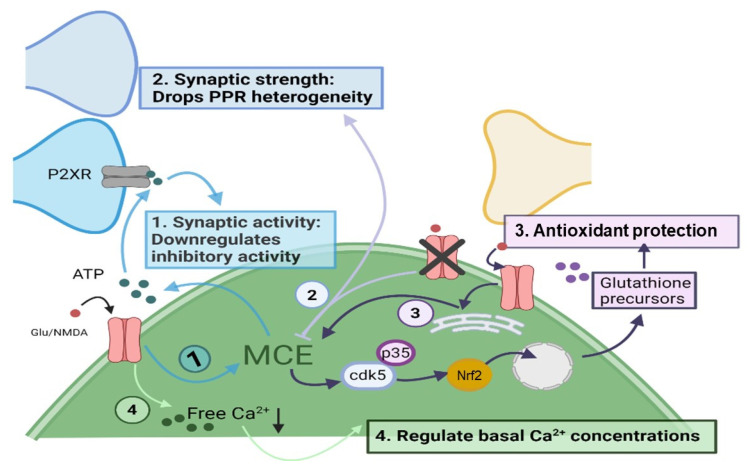
Functional implications of astrocyte NMDA receptors. The following may occur as a result of Antioxidant protection NMDAR activity, possibly via astrocyte calcium events: (1) Modulation of synaptic activity; ATP gliotransmission is evoked that acts on presynaptic P2XRs and thus downregulates inhibitory activity [130]. (2) Regulation of synaptic strength: reduced astrocyte NMDAR expression decreases the paired-pulse ratio variability [49,139] (3) Protection of neurons against antioxidant stress; NMDAR activation upregulates expression of cdk5/p35 that promotes expression of glutathione precursors through Nrf2 [121]. (4) Regulation of basal astrocyte Ca^2+^ concentrations, which can define MCEs characteristics such as amplitude and peak frequency [26,27].

Astrocyte NMDA receptors have functional roles in maintaining astrocyte Ca^2+^ stores [131], antioxidant protection [121], gliotransmission [130], and the regulation of synaptic strength (Figure 3) [49]. First, pharmacological intervention during theta-burst cortical stimulation suggests that NMDA receptor activity decreases free Ca^2+^ in astrocytes through elevation of store uptake [131]. Thus, NMDA receptors may regulate basal astrocyte Ca^2+^ concentrations, which has implications for Ca^2+^ microdomain activity and their dynamics [26,27]. Second, NMDA-induced somatic Ca^2+^ transients in cultured cortical astrocytes upregulate the Cdk5/Nrf2 pathway, a key regulator of genes for cell antioxidant machinery [121]. This increases the release of glutathione precursors from astrocytes, which are used by nearby neurons to synthesize glutathione, an important antioxidant. Therefore, activation of astrocytic NMDA receptors may contribute to neuronal protection against oxidative stress. NMDA receptor antagonists cause neurotoxicity [138], and conceivably, a loss of astrocyte NMDA receptor activity by receptor blockade, may remove their antioxidant effects, contributing to neuronal damage. Third, cultured cortical astrocytes release ATP in response to NMDA treatment, which may decrease synaptic inhibition of pyramidal neurons in the cortex [130]. 

As discussed in Section 2, astrocytic ATP modulates both autistic-like and depressive-like behaviors in mice [54,55], and ATP-derived adenosine regulates basal synaptic transmission [39] and upregulates somatostatin interneuron synaptic activity [40]. Taken together, gliotransmitter release evoked by astrocyte NMDA receptor activity has the potential to alter nearby neuronal responses or play a role in behaviour. If NMDA receptors induce fast onset Ca^2+^ events in astrocytes, then gliotransmitter release may happen in a temporal realm that can rapidly tune synaptic activity. Finally, astrocyte NMDA receptors may also regulate synaptic strength in the hippocampus by maintaining paired-pulse ratio heterogeneity, which is seen between two presynaptic neurons that target the same postsynaptic cell [49]. Paired-pulse ratio heterogeneity was reduced when: (a) astrocytes were patch-loaded with BAPTA, (b) astrocytes were patch-loaded with NMDA receptor antagonist, MK-801, or (c) the GluN1 subunit of NMDAR was knocked out specifically in astrocytes. This strongly underlines the importance of astrocytic Ca^2+^ transients and astrocytic NMDA receptor signalling in diversifying the presynaptic strengths and potentially elevating the network dynamics of dendrites [49]. This type of regulation may be highly specialized within specific circuits. For example, the diversity of presynaptic strengths in the stratum radiatum of the hippocampus is specifically maintained by astrocyte NMDA receptors containing the GluN2C subunit [139]. While there is some evidence of a functional role for astrocyte NMDA receptors regarding gliotransmission, antioxidant protection, and synaptic modulation, further studies that selectively target NMDA receptors, such as knock-out of the GluN1 subunit in astrocytes, will advance the concepts of Ca^2+^ signalling mediated by these receptors and their physiological roles.

### 3.2. P2X Receptors

#### 3.2.1. Astrocyte P2X Receptor Expression

Astrocytes express ionotropic P2X purinergic receptors (Figure 2), likely composed of heterotrimeric P2X_1/5_ [140] or homotrimeric P2X_7_ subunits [1,141]. These ligand-gated ion channels bind synaptic ATP and conduct Ca^2+^, K^+^, and Na^+^ into the cell. The subunit composition confers ATP binding affinity and Ca^2+^ permeability [1,142,143]. P2X_7_ receptors are only activated by high extracellular ATP levels and have been linked to pathology and astrocyte reactivity [144,145]. Therefore, P2X_1/5_, with its higher affinity for ATP and good Ca^2+^ permeability, is more likely to be involved in astrocyte MCEs, particularly with a fast onset during local circuit activity. So far, the contribution of P2X_1/5_ activity to astrocyte MCEs has not been explored with GECIs, but P2X activation causes astrocyte Ca^2+^ transients (primarily somatic) in brain slices and acutely isolated astrocytes, as measured with Ca^2+^ dyes [109,146].

#### 3.2.2. Functional Roles of Astrocyte P2XRs

Coincidently, astrocyte P2X receptor activation enhances purinergic signalling in different brain regions. In the cortex, astrocyte P2X receptors increase ATP release [147], which modulates nearby synapses. Further, ATP release by astrocytes in the brain stem is evoked by decreased pH, and propagated and amplified by neighbouring astrocytes via P2X receptor activation [148]. This induces the respiratory reflex and increases the breathing rate [148]. Additionally, astrocyte P2X_1_ receptors have been linked to endfoot Ca^2+^ transients and capillary dilation during neurovascular coupling, suggesting that these ionotropic receptors induce the release of vasoactive molecules that specifically act on capillaries and not arterioles [146]. Astrocyte P2X receptor activity also decreases with age [132,147], which leads to an increase in inhibitory and a decrease in excitatory neurotransmission [147] as well as impaired LTP [149]. These effects can be mitigated in aged mice through environmental enrichment and caloric restriction [147], which has important implications for the plasticity of astrocyte activity, and the modulation of synaptic transmission and neurovascular coupling by astrocytes via purinergic signalling. Further functional roles of astrocyte P2X receptors will be identified by future studies selectively targeting these receptors by genetic approaches (i.e., astrocyte P2X receptor knockouts).

### 3.3. Nicotinic Receptors

#### 3.3.1. Astrocyte Nicotinic Receptor Expression

Nicotinic receptors are pentameric ionotropic acetylcholine receptors that conduct Ca^2+^, Na^+^ and K^+^ and are made up of 16 possible subunits. Astrocytes express homomeric alpha-7 nicotinic acetylcholine receptors (α7nAChRs; Figure 2), and activation of these astrocyte receptors in culture or in hippocampal slices induces intracellular Ca^2+^ transients [150,151]. Based on their subunit composition, α7nAChRs have high Ca^2+^ permeability, but are rapidly deactivated [152], suggesting they may cause more brief Ca^2+^ events in astrocytes. α7nAChRs Ca^2+^ transients are further amplified in astrocytes by Ca^2+^ release from intracellular Ca^2+^ stores through ryanodine receptors [150]. At this point, α7nAChR activation has not yet been linked to localized astrocyte MCEs.

#### 3.3.2. Functional Roles of Astrocyte Nicotinic Receptors

Functionally, astrocyte α7nAChRs activation in the hippocampus by acetylcholine from medial septal projections induces D-serine release, leading to nearby neuronal NMDA receptor modulation [153]. This is notably activated by wakeful acetylcholine levels and oscillates throughout the day, creating a rhythmic pattern of gliotransmission [153]. Nicotinic receptor activation also induces morphological changes in the processes of cultured astrocytes [154], which has implications for perisynaptic astrocyte process coverage and remodeling in intact circuits. Finally, α7nAChRs activation in cultured astrocytes upregulates Nrf2 antioxidant genes during inflammation, suggesting astrocyte nAChRs are neuroprotective and decrease oxidative stress [155]. Future studies with GECIs and specific genetic approaches to selectively target astrocyte α7nAChRs will further determine the role of nicotinic receptors in astrocyte physiology and MCE dynamics.

### 3.4. Na^+^-Ca^2+^ Exchanger

#### 3.4.1. Astrocyte Na^+^-Ca^2+^ Exchanger Expression

Astrocytes express the Na^+^/Ca^2+^ exchanger (NCX), which has an important role in buffering intracellular Ca^2+^ in exchange for Na^+^ influx (Figure 2) [156,157,158]. Increased intracellular Na^+^ levels can cause NCX to reverse direction where it brings extracellular Ca^2+^ in for Na^+^ efflux and this creates Ca^2+^ events in astrocytes [115,125]. Importantly, NCX is primarily confined to fine peri-synaptic astrocyte processes where it is frequently localized with the Na^+^/K^+^ ATPase and glutamate transporters that work together to take up glutamate and buffer ion gradients [159,160,161]. This creates an insular compartment for Ca^2+^ and Na^+^ signalling that is potentially ideal for the localization of MCEs [158].

Several possible mechanisms increase intracellular astrocyte Na^+^ and trigger NCX reversal, including (a) glutamate activation of Na^+^-permeable ionotropic kainate or NMDA receptors [125,162,163], (b) excitatory amino acid transporters which utilize the extracellular Na^+^ gradient to drive synaptic glutamate uptake [14,164,165], or (c) GABA transporter (GAT-3), which also conducts Na^+^ into the cell during GABA uptake [46,166]. Ca^2+^ events due to NCX reversal may also trigger Ca^2+^-induced Ca^2+^ release from intracellular Ca^2+^ stores, suggesting NCX reverse function amplifies agonist-induced Ca^2+^ events in astrocytes [164,166].

#### 3.4.2. Functional Roles of Astrocyte NCX Reversal

Astrocyte NCX reversal and increased cellular Ca^2+^ may evoke gliotransmitter release, such as glutamate [167,168], ATP/adenosine [46], and homocysteic acid, the endogenous ligand for NMDA receptors [133]. An increase in extracellular adenosine as a result of GABA uptake and NCX reversal suppresses glutamatergic signalling by activating presynaptic adenosine receptors [46]. This is one way that NCX activity may cause astrocyte Ca^2+^ transients and regulate excitatory transmission. While a number of studies have attempted to model the contribution of NCX to astrocyte MCEs in fine processes [169,170,171], further work is required using GECIs to determine the role of NCX in astrocyte MCE formation and temporal dynamics, particularly regarding the rapid modulation of physiologically active circuits.

### 3.5. Voltage-Gated Calcium Channels

Astrocytes also express voltage-gated Ca^2+^ channels (VGCCs) [172,173,174], although at lower levels than neurons [175]. These channels open during membrane depolarization, permitting the influx of extracellular Ca^2+^. VGCCs do not contribute to spontaneous MCEs [176], but they are activated following astrocyte depolarization due to extracellular K^+^ uptake [174], as well as possible astrocyte NMDA receptor activity [49]. Thus, astrocyte VGCC activity is likely evoked during circuit stimulation when synaptic K^+^ and glutamate accumulate. Functionally, astrocyte VGCCs induce the release of glutamate [174] and possibly other gliotransmitters that maintain the heterogeneity of presynaptic strengths [49]. However, evidence linking VGCC activity with astrocyte MCEs and other functional roles of astrocytes is lacking.

## 4. Conclusions

Astrocytes have localized, rapid fluctuations in intracellular Ca^2+^ that grant them the potential to quickly regulate local processes such as synaptic activity and blood flow. By aiding the integration of information at multiple synapses and among different neuronal types in brain circuits, astrocytes may impact neuronal activity at the population level, thereby contributing to information processing and animal behaviour. Future experiments should focus on GECI tools to better identify MCEs, particularly with a fast onset during nearby synaptic activity. Additionally, pathways of extracellular calcium influx outlined here have important implications for astrocyte calcium physiology and are gaining interest in the field. Approaches to selectively target these pathways will help to better understand their contribution to rapid onset astrocyte MCEs and their functional relevance regarding neuronal network activity.

## Figures and Tables

**Table 1:** Evidence of astrocyte iGluR-mediated Ca^2+^ activity from Ca^2+^ imaging in cell culture studies. The concentration of NMDA is noted when over (100 µM) or under (20 µM) the toxic concentration (50 µM). ✓ and ✕ show the presence or absence of function receptors in each study. Agonists: Glutamate (Glu), kainate (KA), quisqualate (QA), Glycine (Gly), *N*-methyl-D-aspartate (NMDA), α-amino-3-hydroxy-5-methyl-4-isoxazolepropionic acid (AMPA).

Culture Preparation	Pharmacology	Receptor Functionality	Reference
Rat cortical astrocytes 14–21 days in culture	Agonist: Glu, KA NMDA (100 µM)	✓Kainate/AMPA receptors ✕ NMDARs	Pearce et al., 1986. [113]
Rat hippocampal astrocytes 1–3 weeks in culture	Agonist: Glu, QA, KA, Gly, NMDA (100 µM) Blocker: Ca^2+^-free saline aCSF (EGTA)	✓Kainate/AMPA receptors ✕ NMDARs	Cornell-Bell et al., 1990. [114]
Rat cortical astrocytes 4–9 weeks in culture	Agonist: Glu, KA, QA NMDA (100 µM) Blocker: kynurenic acid, Ca^2+^-free saline (EGTA)	✓Kainate/AMPA receptors ✕ NMDARs	Jensen et al., 1990. [115]
Rat hippocampal astrocytes 2–4 weeks in culture	Agonist: KA, AMPA, Gly, NMDA (100 µM)	✕ iGluRs (no Ca^2+^ permeable forms)	Cai et al., 1997. [118]
Rat cerebellar, hippocampal, and cortical astrocytes 10–20-days in culture	Agonist: QA, AMPA Antagonist: CNQX	✓ AMPARs	Glaum et al., 1990. [122]
Rat cortical astrocytes 12–14-days in culture	Agonist: Glu, NMDA (20 µM) Antagonist: MK801, CNQX	✕ Kainate/AMPA receptors ✓ NMDARs	Jimenez-Blasco et al., 2015. [121]
Rat cerebellar astrocytes 4 weeks in culture	Agonist: Glu/Hypoxia Antagonist: CNQX	✓ AMPARs	Kou et al., 2019. [123]

**Table 2:** Evidence of iGluR-mediated Ca^2+^ activity from Ca^2+^ imaging in ex vivo brain slices or acutely isolated astrocytes. Bath or pipette application of drugs are indicated, which affects cell-type specificity. ✓ and ✕ show the presence or absence of function receptors in each study.

Astrocyte Preparation	iGluR Pharmacology	Receptor Functionality	Reference
Hippocampal slices from 10–13-days-old rats	Bath-applied	✓ iGluRs (type not specified)	Porter et al., 1996. [92]
Hippocampal slices from 8-day-old rats	Bath-applied	✓ NMDARs	Pasti et al., 1997. [93]
Hippocampal slices of 31–38-days-old rats	Bath-applied	✓AMPARs ✕ NMDARs	Shelton et al., 1999. [127]
Cortical slice from 1–4-week-old GFAP-EGFP mice	Patch-applied	✓ NMDARs	Schipke et al., 2001. [128]
Hippocampal slice from 10–18-month-old GFAP-EGFP mice	Patch-applied	✓ NMDARs	Serrano et al., 2008. [129]
Optic nerve isolated from 15–30-day-old- GFAP-EGFP mice	Bath-applied	✓ AMPARs ✓ NMDARs	Hamilton et al., 2008. [124]
Brain slices and acutely isolated cortical astrocytes from 3-month-old GFAP-EGFP mice	Patch-applied	✓NMDARs	Palygin et al., 2010. [109]
Neocortical slice from 1–21-months-old GFAP-EGFP mice	Patch-applied	✓AMPAR ✓NMDAR	Lalo et al., 2011. [132]
Cortical astrocytes isolated from adult GFAP-EGFP mice	Patch-applied	✓ NMDAR	Palygin et al., 2011. [108]
Cortical astrocytes isolated from adult mice	Bath-applied	✓ NMDAR	Lalo et al., 2014. [130]
Brain slices and acutely isolated cortical astrocytes from 35–59-day-old GFAP-EGFP mice	Bath-applied	✓ NMDARs	Dzamba et al., 2015. [110]
Olfactory bulb slice from 14–21-day-old Aldh1l1-eGFP mice	Bath-applied	✓ AMPARs ✓ NMDARs	Otsu et al., 2015. [28]
Somatosensory neocortex slice from 21–30-day-old-rats	Patch-applied	✓ NMDARs	Mehina et al., 2017. [131]
Olfactory bulb slice from 8–12-day-old GFAP-EGFP and GLAST-CreERT2-GCaMP6s^fl/fl^ mice	Bath-applied	✓ AMPARs	Droste et al., 2017. [134]

## Data Availability

Not applicable.
